# CauseHSI: Counterfactual-Augmented Domain Generalization for Hyperspectral Image Classification via Causal Disentanglement

**DOI:** 10.3390/jimaging12020057

**Published:** 2026-01-26

**Authors:** Xin Li, Zongchi Yang, Wenlong Li

**Affiliations:** College of Computer Science and Technology, China University of Petroleum (East China), Qingdao 266580, China; lix@upc.edu.cn (X.L.); z23070032@s.upc.edu.cn (W.L.)

**Keywords:** hyperspectral image, cross-scene, counterfactual generation, causal disentanglement

## Abstract

Cross-scene hyperspectral image (HSI) classification under single-source domain generalization (DG) is a crucial yet challenging task in remote sensing. The core difficulty lies in generalizing from a limited source domain to unseen target scenes. We formalize this through the causal theory, where different sensing scenes are viewed as distinct interventions on a shared physical system. This perspective reveals two fundamental obstacles: interventional distribution shifts arising from varying acquisition conditions, and confounding biases induced by spurious correlations driven by domain-specific factors. Taking the above considerations into account, we propose CauseHSI, a causality-inspired framework that offers new insights into cross-scene HSI classification. CauseHSI consists of two key components: a Counterfactual Generation Module (CGM) that perturbs domain-specific factors to generate diverse counterfactual variants, simulating cross-domain interventions while preserving semantic consistency, and a Causal Disentanglement Module (CDM) that separates invariant causal semantics from spurious correlations through structured constraints under a structural causal model, ultimately guiding the model to focus on domain-invariant and generalizable representations. By aligning model learning with causal principles, CauseHSI enhances robustness against domain shifts. Extensive experiments on the Pavia, Houston, and HyRANK datasets demonstrate that CauseHSI outperforms existing DG methods.

## 1. Introduction

Hyperspectral image (HSI) classification exploits the rich spectral-spatial characteristics across hundreds of contiguous bands, enabling fine-grained material recognition and showing great potential in precision agriculture [1], environmental monitoring [2], and geological surveying [3]. With the development of deep learning, convolutional neural networks (CNN) [4,5,6] and Transformers [7,8,9] have significantly improved single-scene HSI classification performance by learning nonlinear spectral-spatial representations. In addition, HSI-specific techniques such as band selection [10] and hyperspectral unmixing [11] help reduce spectral redundancy and noise, further improving robustness and efficiency for downstream classification. However, most existing methods rely on the assumption of independent and identically distributed (i.i.d.) data between training (source) and testing (target) sets, an assumption that often fails in real-world cross-scene applications. Variations in different sensors, atmospheric conditions, illumination, and seasonal dynamics [12,13] lead to substantial domain shifts between scenes, severely degrading model performance when deployed on unseen domains.

To mitigate this domain shift, Domain Generalization (DG) methods have been used to learn domain-invariant features from the source domain(s) only, thereby enabling zero-shot transfer to unseen target domains. DG has made significant progress in various areas such as computer vision and natural language processing. Existing DG approaches can be broadly categorized into three groups: data manipulation, representation learning and learning strategy [14,15]. Data manipulation methods, such as data augmentation [16] and data generation [17,18], enhance diversity and quantity of input data to improve generalization. Representation learning, the mainstream of DG research, mainly follows two directions. One is domain-invariant representation learning, which seeks to learn representations that are invariant across domains [19,20]. The other is feature disentanglement, which attempts to decompose features into domain-invariant and domain-specific components [21,22]. Learning strategies adopt general training paradigms like meta-learning [23,24] and self-supervised learning [25,26] to improve robustness and reduce domain dependency.

However, DG in the field of HSI classification remains relatively under-explored. Currently, research on DG in HSI mainly focuses on the single-source domain generalization setting. This focus arises from the practical challenge that it is difficult and costly to obtain labeled data from multiple domains in HSI, making single-source generalization both necessary and meaningful. Due to the limitation of having only a single labeled domain, existing methods commonly adopt data manipulation techniques to generate pseudo-domains [27,28,29], thereby simulating domain shifts between different environments. Based on this, two major strategies have emerged to enhance generalization: one leverages contrastive learning and adversarial training to obtain domain-invariant representations [30,31], while the other focuses on feature disentanglement to separate domain-shared and domain-specific features [32,33].

While these efforts provide valuable insights, further advancements are needed to fully address the challenges posed by complex and diverse domain shifts in HSI data. In this work, we introduce a causal perspective to understand and address the generalization problem under single-source DG settings. Specifically, we regard different sensing scenes as interventions on a shared physical system, and focus on two core challenges that hinder robust generalization across such interventional shifts.

Challenge 1: Learning stable representations from limited interventional diversity. Under the single-source DG setting, models are trained on data collected from a single sensing scene, which corresponds to a specific intervention on the underlying physical system. This lack of interventional diversity makes it difficult for the model to capture causal mechanisms that are invariant across scenes. Consequently, the learned representations may overfit to scene-specific patterns and fail to generalize to unseen domains.

Challenge 2: Eliminating spurious correlations that confound semantic prediction. In source domains, certain non-semantic factors—such as background textures, atmospheric conditions, or sensor-specific artifacts—may spuriously correlate with class labels. These confounders can mislead the model into learning shortcuts that do not hold in unseen domains, leading to degraded generalization performance. The core difficulty lies in identifying and separating truly causal semantic features from such entangled spurious ones.

**Figure 1:**
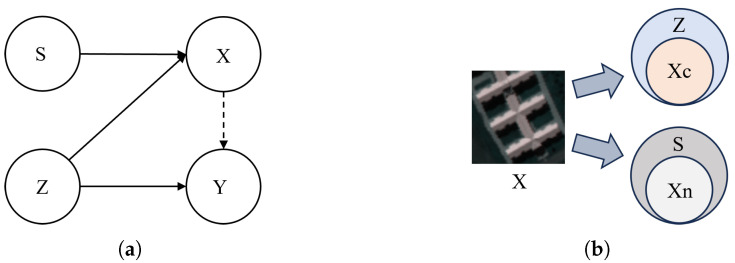
(**a**) SCM of the image generation process. *Z*, *S*, *X* and *Y* denote the latent physical properties, sensing scene, observed image, and semantic label, respectively. (**b**) Illustration of representation disentanglement. $X_{c}$ and $X_{n}$ denote the causal features and non-causal features, respectively.

To formally characterize domain shifts in HSI, we adopt a Structural Causal Model (SCM) [34] to model the data-generating process, as illustrated in Figure 1a. The latent physical properties (Z) determine the semantic label (Y), while the observed image (X) is influenced by both the latent properties (Z) and the sensing scene (S). Scene variations introduce domain-specific artifacts in *X*, but do not affect the intrinsic semantics *Y*.

Under a single-source setting ($S=s_{0}$), the model only observes limited interventional diversity, leading to Challenge 1. Moreover, since *X* encodes both semantic (*Z*-related) and spurious (*S*-related) components, their entanglement leads to Challenge 2. To illustrate this, Figure 1b shows how *X* can be decomposed into causal features $X_{c}$ (derived from *Z*) and non-causal features $X_{n}$ (originating from *S*), both of which may correlate with *Y* in the training domain. Such spurious correlations may bias predictions when deployed under unseen scenes. In light of these issues, our goal is two-fold: simulate diverse interventions *S* to encourage stable feature learning; and disentangle *X* into $X_{c}$ and $X_{n}$ to isolate invariant semantic cues from scene-dependent noise.

To solve the aforementioned challenges and achieve our goal of robust generalization under single-source domain settings, we propose CauseHSI, a causality-inspired framework composed of two key components: the Counterfactual Generation Module (CGM) and the Causal Disentanglement Module (CDM).

To tackle Challenge 1, CGM adopts a causal perspective to simulate interventional diversity under the single-source setting. Based on the SCM in Figure 1a, where domain shifts stem from variations in the sensing condition *S*, we approximate interventions $\mathrm{do}(S=s^{\prime })$ (do-operator is a mathematical operator for intervention.) [35] through controlled perturbations of the observed image *X* in the frequency domain. This approximation does not aim to reproduce exact physical sensing processes, but instead provides an operational means to emulate plausible sensing-induced variations while preserving semantic consistency. Motivated by findings that domain-specific artifacts tend to concentrate in extreme frequency bands [28,36,37,38], we decompose the frequency representation of *X* and apply structured Gaussian noise to low and high frequencies, which serve as a practical approximation of scene-sensitive variations, while preserving mid-frequency components that are relatively more robust to sensing changes and thus act as scene-robust carriers of semantic information. To further ensure semantic fidelity, CGM incorporates two complementary mechanisms: injecting the central spectral signature to retain class-discriminative cues, and applying mild spatial randomization to enrich local diversity without disrupting spatial structure. In addition, a style-controlled discrepancy loss explicitly constrains the magnitude of perturbations, preventing excessive deviations that could compromise label consistency. Through these constrained interventions in the frequency, spectral, and spatial domains, CGM generates counterfactual samples [39], namely synthetic data instances derived by perturbing domain-specific factors while preserving class-discriminative semantics. These counterfactual samples effectively expand the range of sensing conditions beyond the single-source domain, thereby enriching interventional diversity and alleviating the generalization limitations described in Challenge 1.

To address the second challenge, we propose the CDM, guided by three principles: causal independence, cross-domain consistency, and semantic completeness. The detailed theoretical foundation is elaborated in Section 3.3. CDM adopts a dual-branch structure to isolate causal and non-causal features based on marginal independence assumptions. To enhance semantic completeness and ensure consistency across domains, we design a Causal Reassembly Module (CRM), which reconfigures features in the frequency domain by decomposing non-causal features into high- and low-frequency components and recombining them with causal representations. This reconstruction enforces complementary constraints that encourage the disentangled causal branch to capture more authentic, invariant semantics.

In summary, CauseHSI enables robust generalization under single-source settings by simulating interventional shifts through controlled frequency perturbations and disentangling domain-invariant causal semantics via principled architectural constraints, all from a causality-inspired perspective. The major contributions of the proposed method are summarized as follows.

1. We present a novel perspective for cross-scene HSI classification under single-source DG by framing the problem within a SCM, which explicitly accounts for sensing-induced interventions and their effects on feature entanglement.
2. To simulate unseen domain shifts, we introduce CGM, which perturbs extreme frequency components of HSI data in a controlled manner, generating semantically consistent counterfactual samples. This module exposes the model to diverse sensing conditions, enhancing robustness.
3. We propose CDM to explicitly disentangle causal and non-causal representations using a dual-branch architecture and a causal reassembly mechanism. This enables the model to isolate invariant semantic cues from domain-specific artifacts.

The remainder of this paper is organized as follows. Section 2 introduces related works pertinent to this study. Section 3 elaborates on the proposed methodology in detail. Section 4 reports experimental results and comparative analyses. Finally, Section 5 concludes the paper with a summary of the proposed approach and future directions.

## 2. Related Work

### 2.1. Single-Source Domain Generalization

DG aims to learn a model from one or multiple source domains that generalizes well to unseen target domains with distinct distributions. Among various DG settings, single-source DG presents a particularly challenging scenario, as models must achieve generalization without exposure to target domain data or multiple source distributions. This setting is especially relevant in hyperspectral remote sensing, where acquiring data from multiple domains is often impractical due to high labeling costs and acquisition constraints.

In single-source DG, the core challenge lies in mitigating the overfitting of models to source-specific characteristics while extracting domain-invariant features that can generalize across diverse, unseen environments. The lack of cross-domain supervision exacerbates this problem, making it difficult for models to disentangle semantic content from domain-specific noise.

To overcome this, recent works have explored domain diversification within the source domain itself, particularly through generative models that synthesize pseudo-domains to mimic unseen distributions. SDEnet [30] introduces an encoder-randomization-decoder framework that incorporates spatial and spectral perturbations to generate diverse domain variants, further enhanced by supervised contrastive learning and adversarial objectives. LLURNet [31] adopts a locally linear unbiased randomization approach using symmetric autoencoders to embed style variations while preserving semantic consistency, with contrastive regularization to stabilize training. Beyond random perturbations, D3Net [40] utilizes a domain-adversarial generator and dual-branch discriminators to extract domain-agnostic features, offering a more structured alternative to handcrafted augmentations. ISDGS [41] extends this line of work by introducing semantic-style covariance generation and spatial shuffling mechanisms, jointly optimized under a dual-sampling adversarial contrastive framework to enhance semantic preservation and domain robustness.

These methods demonstrate the effectiveness of expanding the source domain through synthetic variations to simulate domain shifts and enhance generalization. However, balancing domain perturbation with semantic and spectral integrity remains an open challenge, motivating further innovations in structured domain expansion techniques.

In parallel, a number of recent studies have focused on improving HSI classification through more powerful spectral-spatial representation learning rather than explicit domain generalization. For instance, hierarchical transformer architectures enhanced with deformable convolutions and frequency-aware attention mechanisms have been proposed to better capture local homogeneity and long-range dependencies in complex HSI [42]. Other works incorporate context-aware masking and diffusion-guided feature refinement to improve robustness against spectral-spatial perturbations and label scarcity [43]. Additionally, spectral-spatial perception networks tailored for specific applications, such as mineral hyperspectral classification, leverage frequency-domain spatial modeling and fine-grained spectral attention to enhance discrimination among visually similar classes [44]. While these approaches achieve impressive performance under in-domain or task-specific settings, they generally rely on correlation-driven representations and implicitly assume similar data distributions between training and testing, limiting their robustness under cross-scene domain shifts.

### 2.2. Feature Disentanglement

A core challenge in domain generalization is to separate semantic representations from domain-specific factors within visual features. Feature disentanglement addresses this issue by structuring the learned representation space into independent components that reflect distinct underlying attributes—typically, domain-invariant semantics and domain-related variations. This becomes especially important in single-source domain generalization, where models must rely solely on source data or generated variants to learn representations that are robust to unseen domain shifts. By explicitly decoupling domain-specific and domain-invariant factors, feature disentanglement enables more stable semantic learning and improves generalization across diverse target domains.

Recent studies have explored various strategies to integrate feature disentanglement into domain generalization frameworks. In hyperspectral scenarios, FDFSL [32] introduces an orthogonal low-rank decomposition to suppress source-induced bias and fuses heterogeneous spectral information using a multi-order spectral interaction block with positional encoding, enabling more robust few-shot generalization across domains. To address cross-scene domain shifts, DSDGnet [33] progressively separates domain-invariant and domain-specific features through Transformer-based style transfer and disentanglement modules, supported by a domain combination mechanism that reinforces disentanglement accuracy. Similarly, S4DL [45] employs a gradient-guided spectral-spatial decomposition to disentangle domain-specific and domain-invariant representations in hyperspectral data, further incorporating a shift-sensitive adaptive monitor to adjust disentangling intensity according to the magnitude of domain shift. From a causal perspective, an early-branching framework [46] introduces diverging causal and non-causal feature branches after a shared encoder, effectively reducing entanglement and improving semantic robustness through random domain sampling. In contrast to approaches focused solely on domain invariance, the mDSDI framework [47] jointly learns both invariant and specific features using a meta-optimization strategy, leveraging task-relevant domain-specific knowledge to enhance generalization. Additionally, the CSD method [48] decouples classifier weights into common and specific components via linear low-rank decomposition, providing theoretical identifiability of invariant features and improving efficiency by removing domain-specific components during inference.

These studies demonstrate the effectiveness of feature disentanglement in enhancing domain generalization by promoting semantic stability and reducing domain interference. However, there is often an absence of rigorous constraints to ensure that the decomposed features remain semantically complete and consistent across domains, which can lead to information loss or degraded generalization under distribution shifts.

## 3. Methods

**Figure 2:**
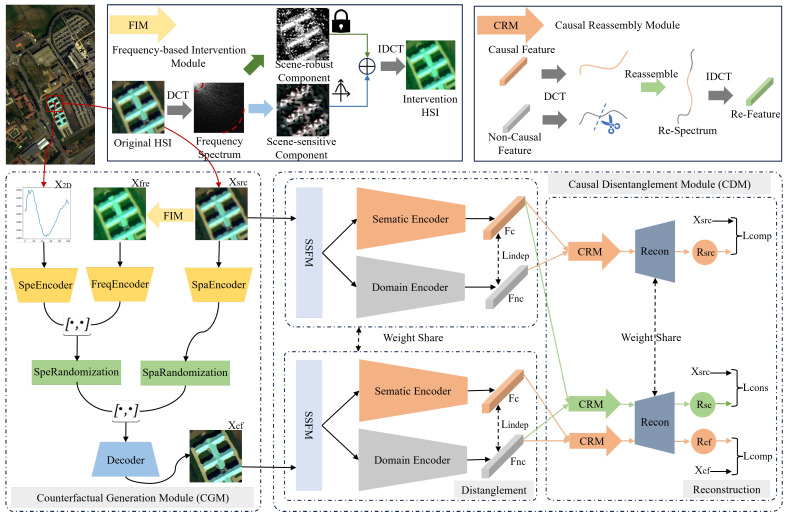
Overall pipeline of the proposed CauseHSI. “[·,·]” means feature concatenation.

The proposed CauseHSI framework, depicted in Figure 2, is composed of two key modules: CGM and CDM. Given source domain hyperspectral samples, CGM generates counterfactual samples by perturbing domain-specific components while preserving semantic content. These counterfactuals simulate plausible distribution shifts, enabling the construction of a source–counterfactual domain pair that reflects potential domain variations. Both original and counterfactual samples are then fed into CDM, which adopts a dual-branch architecture to disentangle features into causal and non-causal components under marginal independence assumptions. A Causal Reassembly Module further refines these representations by enforcing semantic completeness and consistency. Reconstruction and classification losses guide the network to extract domain-invariant causal features. Ultimately, classification is performed based on the causal features, which promote robust generalization across unseen domains.

### 3.1. Causal Formulation of Domain Shifts

To provide a rigorous foundation for our framework, we formalize the SCM underlying HSI classification. As illustrated in Figure 1a, four key variables are considered: latent physical properties *Z*, sensing scene *S*, observed image *X*, and semantic label *Y*. Their relationships are characterized as:

(1) Z∼P(Z),Y∼h(Z),X∼P(X∣Z,S).

Here, P(Z) captures the intrinsic distribution of physical properties, h(·) denotes the semantic generation mechanism that maps physical attributes to class labels through a stable semantic mechanism, and P(X∣Z,S) models the imaging process in which the sensing scene *S* introduces domain-specific variations. This formulation explicitly reflects that *Y* is causally determined by *Z* and is invariant to *S*, while *S* acts as an interventional variable that perturbs the distribution of *X* without affecting the semantic mechanism.

It is worth noting that although *Y* is causally generated from *Z*, the observed image *X* may exhibit spurious statistical correlations with *Y* in the training domain. Such correlations arise from the fixed sensing condition *S* and are illustrated as a dashed arrow from *X* to *Y* in Figure 1a. This dashed edge does not represent a causal influence, but rather an observational dependence induced by domain-specific biases. Under a single-source setting, all training samples are collected under a fixed sensing condition $S=s_{0}$. As a result, the model is exposed to only a limited range of sensing variations, which restricts the diversity of interventional patterns and leads to Challenge 1.

From a causal perspective, the observed hyperspectral image *X* is influenced by both intrinsic semantic factors and extrinsic sensing conditions. Rather than modeling the full physical imaging process, we adopt a functional abstraction to characterize their distinct causal roles. Specifically, we conceptually decompose *X* into a causal component $X_{c}=f\left(Z\right)$, which encodes semantic information determined by latent physical properties, and a non-causal component $X_{n}=g\left(S\right)$, which captures domain-specific variations introduced by the sensing scene:

(2) X=f(Z)+g(S).

This formulation does not imply that the real imaging process is strictly linear or additive. Instead, it serves as a causal abstraction that highlights the separation between invariant semantic factors and scene-dependent perturbations. In practice, complex nonlinear interactions between *Z* and *S* may exist and are implicitly absorbed into the functional representations of f(·) and g(·). The importance of this abstraction lies in enabling a clear causal interpretation of domain shifts, where variations in *S* can be viewed as interventions that alter $X_{n}$ while leaving the semantic mechanism Z→Y invariant. Since *S* remains fixed in the source domain, the non-causal component $X_{n}$ may become spuriously correlated with the label *Y*. Models trained on such data tend to exploit these domain-specific cues, resulting in unstable predictions when encountering unseen sensing conditions. This phenomenon gives rise to Challenge 2.

### 3.2. Counterfactual Generation Module (CGM)

Prior studies [36,49] have shown that the extreme frequency components of images often contain domain-private patterns, which are sensitive to sensing conditions rather than reflecting intrinsic semantics. Building on this observation, we interpret sensing variations as interventions on the path S→X in our SCM (Figure 1a), where the sensing scene *S* introduces domain-specific artifacts into the observed image *X* without altering the underlying semantics *Y*. By perturbing frequency-sensitive components, CGM mimics such scene-induced variations while preserving semantic consistency inherited from the latent properties *Z*. This design allows the generated counterfactual samples to reflect plausible sensing changes, thereby enhancing the robustness of the model under distributional shifts.

**Figure 3:**
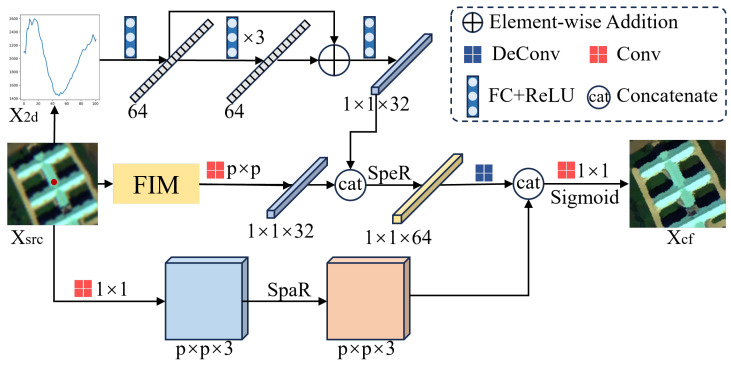
Data processing flow of the CGM. “SpeR” and “SpaR” represent spectral and spatial randomization, respectively.

CGM does not aim to exactly reproduce physical sensing processes or explicitly parameterized sensing variables. Instead, it provides a controlled and operational approximation of plausible sensing variations by selectively perturbing empirically scene-sensitive components while enforcing spectral, spatial, and semantic consistency. This design allows CGM to generate label-consistent counterfactual samples that reflect realistic sensing changes, rather than arbitrary noise injections. The overall architecture of CGM is illustrated in Figure 3, and consists of three coordinated branches.

#### 3.2.1. Frequency-Based Intervention

Given an input hyperspectral patch $X\in R^{p\times p\times C}$, we perform a discrete cosine transform (DCT) on each spectral channel to obtain its frequency representation $FR\in R^{p\times p\times C}$. As shown in Figure 1a, under the structural model X←(Z,S), domain shifts are primarily induced by variations in the sensing condition *S*, while the latent physical properties *Z* remain invariant across scenes. To simulate interventions on the nuisance factor *S* under a fixed source distribution $S=s_{0}$, we perform frequency-domain perturbations as an operational approximation of the structural intervention $\mathrm{do}(S=s^{\prime })$.

Rather than adopting a hard frequency cutoff, we employ a soft frequency weighting strategy in the DCT domain. Frequency components are ordered according to their radial distance from the DC component, and a fixed, smoothly varying weighting profile is applied to modulate different frequency regions. This weighting profile assigns higher perturbation strength to extreme low- and high-frequency components, which are empirically more sensitive to sensing conditions and scene-dependent distortions, while mid-band frequencies receive minimal perturbation and are thus treated as relatively scene-robust semantic carriers.

Formally, the frequency representation is decomposed into two complementary components via soft weighting: a scene-sensitive component $FR_{d}$ and a scene-robust component $FR_{c}$, satisfying $FR=FR_{d}+FR_{c}$. The intervention $\mathrm{do}(S=s^{\prime })$ is approximated by applying stochastic multiplicative perturbations to $FR_{d}$, i.e., ${\tilde{FR}}_{d}=FR_{d}⊙\left(1+\epsilon \right)$ with $\epsilon ∼N(0,\sigma^{2})$, while keeping $FR_{c}$ unchanged. This multiplicative formulation perturbs the magnitude of scene-sensitive frequency components without altering their spatial-frequency structure, thereby avoiding unrealistic artifacts. The intervened frequency representation is then reconstructed as $\tilde{FR}={\tilde{FR}}_{d}+FR_{c}$ and transformed back into the spatial domain via inverse DCT to produce the perturbed image $X_{\mathrm{fre}}$.

Finally, $X_{\mathrm{fre}}$ is encoded by a frequency encoder (FreqEncoder) composed of convolutional blocks to yield a compact frequency-level representation $z_{\mathrm{fre}}\in R^{1\times 1\times 32}$. This frequency-level decomposition provides a controllable and effective proxy for simulating sensing-related variations under limited source diversity.

#### 3.2.2. Spectral Consistency Preservation

To retain class-discriminative information, we enhance the perturbed sample with its central 2D spectral signature. This spectral vector is passed through a SpeEncoder composed of fully connected layers with ReLU activations and a residual connection, producing the spectral embedding $z_{spe}\in R^{1\times 1\times 32}$. The concatenation $\left[z_{fre};z_{spe}\right]$ is further processed by a spectral-level randomization module [30], producing a fused feature $z_{g}\in R^{p\times p\times 64}$ that preserves spectral integrity while introducing controlled variability.

#### 3.2.3. Spatial Style Perturbation

In parallel, we extract spatial features $z_{spa}\in R^{p\times p\times 3}$ from the original patch using SpaEncoder, a shallow CNN. To increase spatial diversity while maintaining structural coherence, we apply Adaptive Instance Normalization (AdaIN):

(3) $$\mathrm{AdaIN}\left(z,\mu^{\prime },\sigma^{\prime }\right)=\sigma^{\prime }\left(\frac{z-\mu }{\sigma }\right)+\mu^{\prime },$$

where (μ,σ) are the per-channel statistics of *z*, and $\left(\mu^{\prime },\sigma^{\prime }\right)$ are randomly sampled from other spatial features within the batch. This transformation perturbs spatial styles without disrupting semantic layout.

The outputs from the spectral and spatial branches are concatenated and passed through a Decoder to produce the final counterfactual sample $X_{\mathrm{cf}}$. To ensure semantic alignment while promoting stylistic diversity, we introduce a style-controlled discrepancy loss:

(4) $$\begin{array}{cc} L_{\mathrm{control}} & =\alpha \cdot ∥\mathrm{Gram}\left(z_{f}\right)-\mathrm{Gram}\left({\tilde{z}}_{f}\right){∥}_{2}^{2}\\ \; & \;+\beta \cdot \left[\max(0,\tau_{\min}-∥z_{f}-{\tilde{z}}_{f}{∥}_{2})\right.\\ \; & \;\left.+\max(0,∥z_{f}-{\tilde{z}}_{f}{∥}_{2}-\tau_{\max})\right], \end{array}$$

where $z_{f}$ denotes the style feature extracted from *X*, and ${\tilde{z}}_{f}$ represents the style feature extracted from the counterfactual sample $X_{cf}$. The first term ensures a sufficient style gap via Gram matrix distance, and the second term constrains the perturbation strength within a controlled range $\left[\tau_{\min},\tau_{\max}\right]$.

Through this controlled intervention in the frequency, spectral, and spatial spaces, CGM generates label-consistent counterfactuals that serve as effective augmentations for improving the model’s generalization across unseen domains.

### 3.3. Causality Feature Disentanglement: Theoretical Foundation

Inspired by causal inference theory, we propose a disentanglement approach that distinguishes causal features from non-causal features. This separation is crucial for learning representations that generalize across unseen domains by emphasizing invariant causal mechanisms. However, identifying causal and non-causal features solely from observational data is inherently challenging, especially in the absence of additional assumptions or constraints. It is important to clarify that we do not claim theoretical identifiability of causal and non-causal factors from single-source observational data alone. Indeed, in classical causal inference, such disentanglement is generally unidentifiable without interventional data, multiple environments, or strong prior assumptions. In our framework, this limitation is explicitly acknowledged and addressed by augmenting the observational setting with counterfactual samples generated by the proposed CGM. These counterfactual samples introduce controlled variations that simulate interventions on non-causal factors, thereby providing additional supervisory signals for learning a practically useful disentanglement. Following the insights from prior works [50,51], introducing appropriate constraints can help approximate the underlying causal structure. In the context of cross-domain generalization, we posit that the disentangled causal and non-causal features should satisfy the following three principles:

1. Causal Independence: Causal and non-causal features should be statistically independent [46].
  (5) $$I(X_{c};X_{n})\approx 0,$$
  where I(·;·) denotes mutual information.
2. Semantic Completeness: The combination of causal and non-causal features should preserve sufficient information for accurate reconstruction or prediction.
  (6) $$H\left(Y|X_{c},X_{n}\right)\approx H\left(Y|X\right),$$
  where H(·) denotes Shannon entropy.
3. Cross-Domain Consistency: Causal features corresponding to the same semantic content should remain invariant across domains.
  (7) $$D\left(P\left(X_{c}^{\left(s\right)}\right),P\left(X_{c}^{\left(t\right)}\right)\right)\approx 0,$$
  where $P(X_{c}^{\left(s\right)})$ and $P(X_{c}^{\left(t\right)})$ denote the distributions of causal features in source and target domains, and D(·) is a distributional distance (e.g., MMD).

These three principles jointly form the theoretical foundation for our causal-inspired disentanglement framework, which we incorporate into the model through architectural design and loss constraints to enable robust generalization under domain shifts.

### 3.4. Independence Constraint: Marginally Independent Representation Decomposition

**Figure 4:**
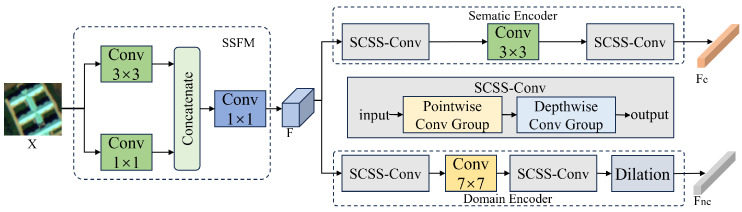
Structure of Disentanglement Module.

To facilitate the disentanglement of causal and non-causal features, we adopt the marginal independence assumption, which posits that the two factors should be statistically independent. This assumption is widely used in causal representation learning and domain generalization [46,50,52], helping to prevent spurious correlations between invariant semantics and domain-specific variations. To implement this assumption, we design a dual-branch network inspired by the early-branching strategy [46], as shown in Figure 4. The network begins with a Spectral-Spatial Fusion Module (SSFM), which extracts low-level spectral-spatial representations from the hyperspectral input. The SSFM integrates channel-wise spectral cues and local spatial structures through parallel convolutions, and fuses them to obtain the shallow feature map $F\in R^{H\times W\times C}$.

Notably, the independence constraint is not imposed solely on source domain observations. Instead, it is jointly applied to source samples and their corresponding counterfactual variants generated by CGM. By exposing the model to paired samples that share semantic content but differ in non-causal factors, the disentanglement process is guided by contrastive supervisory signals that approximate interventional variation. This design alleviates the inherent identifiability ambiguity in single-source observational data and enables the model to separate invariant semantic features from domain-specific variations in a pragmatic manner.

The feature map *F* is fed into two separate encoders: a semantic encoder $E_{c}\left(\cdot \right)$ to extract causal features $F_{c}$ and a domain encoder $E_{nc}\left(\cdot \right)$ to extract non-causal features $F_{nc}$. Inspired by CVSSN [53], each encoder consists of a pointwise convolution group, a depthwise convolution group, and a module tailored for specific semantics: $E_{c}$ emphasizes mid-frequency information via 3×3 convolutions, capturing fine-grained structural and texture patterns that are generally domain-invariant. $E_{nc}$ uses 7×7 convolutions to aggregate broader context and low-frequency trends, while dilated convolutions are added to retain detail and detect high-frequency, domain-specific variations.

To ensure that $F_{c}$ and $F_{nc}$ are statistically independent, we introduce a regularization term based on the Hilbert-Schmidt Independence Criterion (HSIC) [54]. This criterion is a kernel-based statistical dependence measure. Given two variables *P* and *Q* (in our case, $F_{c}$ and $F_{nc}$), the empirical HSIC is computed as:

(8) $$\mathrm{HSIC}\left(P,Q\right)=\frac{1}{{\left(n-1\right)}^{2}}\mathrm{Tr}\left(K_{P}HK_{Q}H\right),$$

where $K_{P},K_{Q}$ are Gram matrices computed using RBF kernels over samples of *P* and *Q*, respectively; $H=I-\frac{1}{n}11^{⊤}$ is the centering matrix; *n* is the batch size.

We define the independence loss as:

(9) $$L_{\mathrm{indep}}=\mathrm{HSIC}\left(F_{c},F_{nc}\right),$$

which is minimized during training to reduce dependency between the two feature branches while promoting better disentanglement.

### 3.5. Completeness and Consistency Constraint: Frequency-Aware Reconstruction Strategy

**Figure 5:**
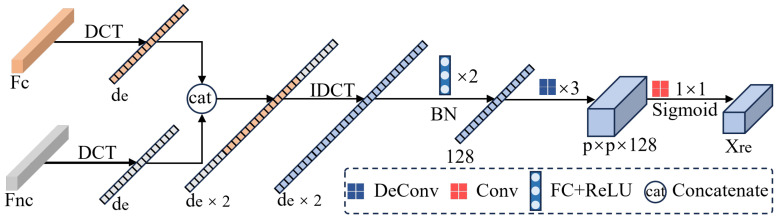
Data processing of reconstruction module. “$d_{e}$” represents the embedding dimension of features.

While marginal independence ensures that causal and non-causal representations do not share statistical dependencies, this alone does not guarantee that they together encode complete and semantically meaningful information. To further enhance disentanglement, we propose a reconstruction process (illustrated in Figure 5) that imposes two complementary constraints—causal completeness and causal consistency—corresponding to Principle 2 and Principle 3.

At the core of this process lies our novel Causal Reassembly Module (CRM), which performs a frequency-aware fusion of causal and non-causal features. Specifically, given the causal feature $F_{c}$ and non-causal feature $F_{nc}$, we first project them into the DCT space: $S_{c}=\mathrm{DCT}\left(F_{c}\right)$, $S_{nc}=\mathrm{DCT}\left(F_{nc}\right)$. We then partition $S_{nc}$ into low-frequency component $S_{nc}^{\mathrm{low}}$ and high-frequency component $S_{nc}^{\mathrm{high}}$. Treating $S_{c}$ as the middle-frequency component, we concatenate the three in frequency order to obtain a reassembled spectrum $S_{re}$, which is then transformed back to the spatial domain via inverse DCT: $F_{re}=\mathrm{IDCT}\left(S_{re}\right)$. This frequency-aware reassembly allows the model to maintain semantic consistency across domains while preserving both coarse and fine structural details.

Finally, the reassembled feature $F_{re}$ is passed through a lightweight decoder $D_{rec}$ to reconstruct the image: $X_{re}=D_{rec}\left(F_{re}\right)$. To ensure faithful reconstruction, we define an $\ell_{1}$ loss between the original image *X* and the reconstructed image $X_{re}$:

(10) $$L_{\mathrm{rec}}={∥X_{re}-X∥}_{1}.$$

To impose the aforementioned constraints, we apply the reconstruction process with different feature combinations as input, enabling the model to learn both the completeness and consistency of disentangled features across domains.

#### 3.5.1. Causal Completeness Constraint (Principle 2)

To ensure that the combined representations $F_{c}$ and $F_{nc}$ capture the full semantic content of the input, we reconstruct both the source image and its counterfactual sample from the reassembled features. The completeness constraint is enforced via the reconstruction losses from both domains:

(11) $$L_{\mathrm{comp}}=L_{\mathrm{rec}}^{\mathrm{src}}+L_{\mathrm{rec}}^{\mathrm{cf}},$$

where $L_{\mathrm{rec}}^{\mathrm{src}}$ denotes the reconstruction loss of the original source image *X*, and $L_{\mathrm{rec}}^{\mathrm{cf}}$ corresponds to that of the counterfactual sample $X_{\mathrm{cf}}$.

#### 3.5.2. Causal Consistency Constraint (Principle 3)

To promote domain-invariant semantics in $F_{c}$, we conduct cross-domain reassembly by combining source domain causal features $F_{c}^{\mathrm{src}}$ with counterfactual-domain non-causal features $F_{nc}^{\mathrm{cf}}$ for reconstruction. The corresponding consistency reconstruction loss is defined as:

(12) $$L_{\mathrm{cons}}^{\mathrm{rec}}={∥D_{rec}\left(\mathrm{CRM}\left(F_{c}^{\mathrm{src}},F_{nc}^{\mathrm{cf}}\right)\right)-X_{\mathrm{cf}}∥}_{1}.$$

In addition, we enforce consistency in the causal feature space directly by minimizing the distance between causal features of the source and counterfactual domains:

(13) $$L_{\mathrm{cons}}^{\mathrm{feat}}={∥F_{c}^{\mathrm{src}}-F_{c}^{\mathrm{cf}}∥}_{2}.$$

The total causal consistency loss is:

(14) $$L_{\mathrm{cons}}=L_{\mathrm{cons}}^{\mathrm{rec}}+L_{\mathrm{cons}}^{\mathrm{feat}}.$$

By systematically satisfying the three core principles, our proposed method achieves a principled disentanglement of causal and non-causal factors in HSI. These principles guide the learning process to isolate scene-invariant, label-relevant representations while suppressing scene-specific variations, thereby improving robustness under domain shifts.

Once the causal representation $F_{c}$ is extracted by the disentanglement module, it is passed through a classification head C(·) to predict class labels. To ensure the discriminative power of causal features extracted from both source and counterfactual images, we supervise the predictions using cross-entropy loss on both:

(15) $$L_{\mathrm{cls}}^{\mathrm{src}}=\mathrm{CE}\left(C\left(F_{c}^{\mathrm{src}}\right),y^{\mathrm{src}}\right),\;L_{\mathrm{cls}}^{\mathrm{cf}}=\mathrm{CE}\left(C\left(F_{c}^{\mathrm{cf}}\right),y^{\mathrm{src}}\right),$$

where CE(·,·) denotes the cross-entropy loss, and $y^{\mathrm{src}}$ is the source domain ground-truth label shared by its counterfactual. The final classification loss is the sum of both:

(16) $$L_{\mathrm{cls}}=L_{\mathrm{cls}}^{\mathrm{src}}+L_{\mathrm{cls}}^{\mathrm{cf}}.$$

### 3.6. Training Phase

In our method, the training procedure involves the joint but alternate optimization of CGM and CDM.

We first optimize the CDM. The objective is to ensure that the extracted features satisfy the three proposed causal principles, while also being discriminative for the final classification task. The total loss $L_{\mathrm{CDM}}$ for optimizing this module is formulated as:

(17) $$L_{\mathrm{CDM}}=\lambda_{1}\left(L_{\mathrm{indep}}+L_{\mathrm{comp}}+L_{\mathrm{cons}}\right)+L_{\mathrm{cls}},$$

where $\lambda_{1}$ is a hyperparameter to balance the contribution of causal loss.

After updating the CDM module, we optimize the CGM with style-controlled discrepancy loss $L_{\mathrm{control}}$ from Equation (4) and counterfactual samples classification loss $L_{\mathrm{cls}}^{\mathrm{cf}}$ from Equation (15). The total CGM loss is defined as:

(18) $$L_{\mathrm{CGM}}=\lambda_{2}L_{\mathrm{control}}+L_{\mathrm{cls}}^{\mathrm{cf}},$$

where $\lambda_{2}$ is a balancing weight. For simplicity, $\lambda_{1}$ in Equation (17) and $\lambda_{2}$ in Equation (4) are set to the same value ($\lambda =\lambda_{1}=\lambda_{2}$).

### 3.7. Causal Scope and Limitations

While the proposed framework is inspired by causal principles, it does not aim to identify the true underlying causal graph of hyperspectral image formation. Instead, CauseHSI adopts a causally motivated structural abstraction in which sensing scenes are treated as interventions that induce distribution shifts on the observed data. Within this formulation, the goal is not causal discovery, but to learn feature representations that are operationally consistent with causal assumptions under scene variations.

Specifically, the proposed causal disentanglement is guided by a set of practical criteria. These criteria serve as inductive biases that discourage the exploitation of scene-specific spurious correlations, rather than as formal guarantees of causal identifiability. Similarly, the counterfactual generation module provides a controlled and operational approximation of plausible sensing variations, instead of an exact simulation of physical sensing processes. As a result, the causal properties enforced by the framework should be understood as consistency-oriented constraints that improve robustness under domain shifts, rather than as theoretically proven causal correctness. This design choice is aligned with common practice in causality-inspired representation learning for domain generalization.

## 4. Experiments

### 4.1. Datasets

To evaluate the generalization performance of our proposed method, we conduct extensive experiments on three widely-used HSI datasets: Pavia, Houston, and HyRANK.

#### 4.1.1. Pavia Dataset

**Figure 6:**
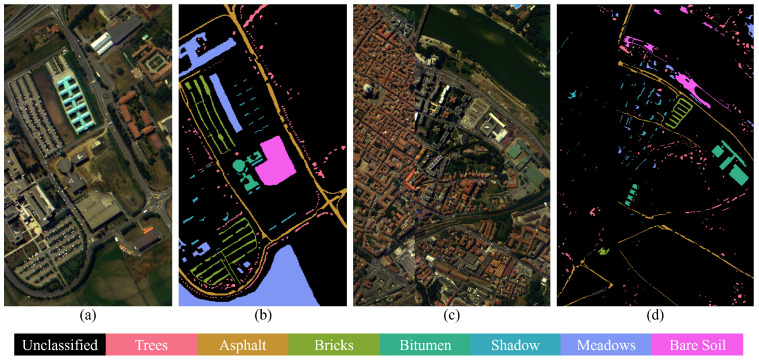
Pseudo-color image and ground truth map of Pavia dataset. (**a**) Pseudo-color image of Pavia University. (**b**) Ground truth map of Pavia University. (**c**) Pseudo-color image of Pavia Center. (**d**) Ground truth map of Pavia Center.

The Pavia dataset consists of two urban scenes: Pavia University and Pavia Center, both captured by the Reflective Optics System Imaging Spectrometer (ROSIS) sensor. The sensor originally records 115 spectral bands within the range of 430–860 nm. After preprocessing to remove noisy bands, Pavia University retains 103 bands, and Pavia Center retains 102 bands. In this study, we use the 102 shared spectral bands between the two scenes. The spatial resolution of both datasets is 1.3 m, with image sizes of 610 × 340 (University) and 1096 × 715 (Center), respectively. We focus on the seven classes that are shared between the two scenes for consistent cross-scene evaluation. The classes and the number of samples are listed in Table 1, and the pseudo-color image with ground truth map is shown in Figure 6.

#### 4.1.2. Houston Dataset

**Figure 7:**
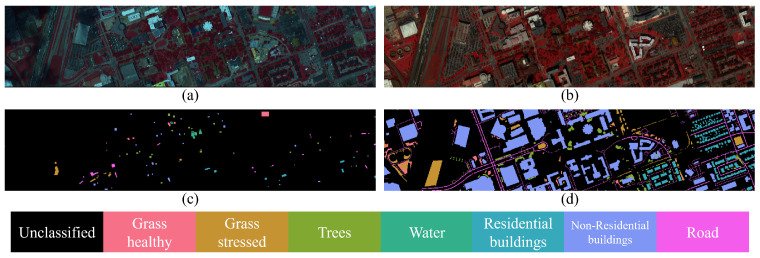
Pseudo-color image and ground truth map of Houston dataset. (**a**) Pseudo-color image of Houston2013. (**b**) Pseudo-color image of Houston2018. (**c**) Ground truth map of Houston2013. (**d**) Ground truth map of Houston2018.

The Houston dataset comprises two subsets: Houston2013 and Houston2018, both acquired over urban areas in Houston, Texas. Houston2013 was captured by the ITRES CASI-1500 sensor (Calgary, AB, Canada) and was provided as part of the IEEE GRSS Data Fusion Contest in 2013. It contains 144 spectral bands spanning 364–1046 nm, with a spatial resolution of 2.5 m and an image size of 349 × 1905. Houston2018, released during the 2018 Data Fusion Contest, features ultrahigh-resolution imagery (0.05 m) with 48 spectral bands over a 2384 × 601 spatial grid. The two subsets are annotated with 15 and 20 land cover categories, respectively. In this study, we adopt the 48 spectral bands and select the seven categories that are common to both subsets to ensure consistency in cross-domain evaluation. The specific number of samples is shown in Table 2. The pseudo-color image and ground truth maps are shown in Figure 7.

#### 4.1.3. HyRANK Dataset

**Figure 8:**
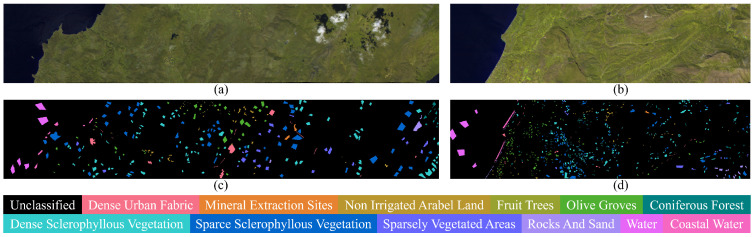
Pseudo-color image and ground truth map of HyRANK dataset. (**a**) Pseudo-color image of Dioni. (**b**) Pseudo-color image of Loukia. (**c**) Ground truth map of Dioni. (**d**) Ground truth map of Loukia.

The HyRANK dataset is derived from Hyperion satellite imagery and includes two scenes: Dioni and Loukia, both located in Greece. Each image has a spatial resolution of 30 m and sizes of 250 × 1376 (Dioni) and 249 × 945 (Loukia), respectively. The Hyperion sensor provides 242 spectral bands in the range of 400–2500 nm, from which 176 bands are retained after removing noisy and water absorption bands. The dataset is labeled with 14 land cover categories, among which 12 consistent classes are selected for training and evaluation in our experiments, as shown in Table 3. The pseudo-color image and ground truth maps are shown in Figure 8.

### 4.2. Implementation Details

All experiments are conducted using the PyTorch (version: 1.8) deep learning framework on a workstation running Ubuntu 20.04.5 LTS with a Linux kernel version of 4.15.0. The hardware configuration includes an Intel(R) Xeon(R) Silver 4210 CPU @ 2.20 GHz and a single NVIDIA GeForce RTX 2080 Ti GPU with 11GB of memory.

We evaluate the performance of our method using four widely adopted metrics in HSI classification: class-specific accuracy, overall accuracy (OA) and the Kappa coefficient (KC).

**Figure 9:**
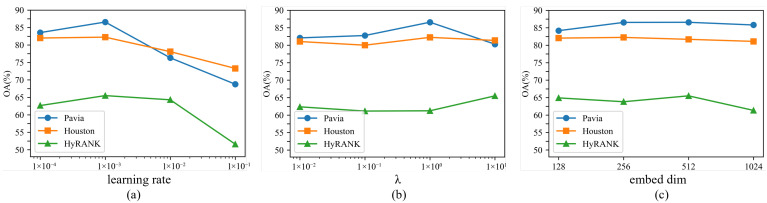
Impact of different parameters on OA across the three datasets. (**a**) learning rate. (**b**) λ. (**c**) embedding dimension.

The training process is configured with a batch size of 256, input patch size 13×13 and 400 training epochs. We apply L2 regularization with a weight decay of $1\times 10^{-4}$. To assess CauseHSI’s sensitivity to key hyperparameters, we analyze three parameters: base learning rate, λ, and embedding dimension ($d_{se}$ in Figure 5), chosen from $\left\{10^{-4},10^{-3},10^{-2},10^{-1}\right\}$, $\left\{10^{-2},10^{-1},10^{0},10^{1}\right\}$, and {128,256,512,1024}, respectively. As shown in Figure 9, the learning rate exhibits consistent behavior across all datasets, with $1\times 10^{-3}$ yielding the best or near-best performance, indicating stable optimization dynamics. For λ, performance varies smoothly over a wide range of values. While the optimal λ differs slightly across datasets, λ=1 remains competitive on all benchmarks, and larger values can further benefit more complex scenes, suggesting that the model does not rely on precise tuning of this parameter. Similarly, the embedding dimension demonstrates a broad plateau of strong performance. Dimensions of 256 and 512 achieve comparable results across datasets, indicating that CauseHSI is not overly sensitive to the specific embedding capacity. Overall, these results confirm that CauseHSI maintains stable performance under reasonable hyperparameter variations, and the dataset-specific settings reflect minor adaptations to data characteristics rather than strict configuration dependencies. Based on the above analysis, we fix the base learning rate to $1\times 10^{-3}$ for all datasets. λ is selected from 1, 10, where λ=1 yields strong performance on Pavia and Houston, while a larger value (λ=10) is adopted for HyRANK to better accommodate its higher scene complexity. The embedding dimension is chosen as 512 for Pavia and HyRANK, and 256 for Houston.

### 4.3. Results and Analysis

To comprehensively evaluate the effectiveness of our proposed method in cross-scene HSI classification, we compare it against a wide range of representative and state-of-the-art approaches. Specifically, we include several recent DG methods tailored for HSI tasks, including SDEnet [30], FDGNet [28], S2ECNet [29], D3Net [40], and ISDGS [41], which are designed to learn scene-invariant representations under unseen target domains. To provide additional reference points, we also include two representative Domain Adaptation (DA) methods, DSAN [55] and TSTnet [56], for supplementary comparison. Additionally, we include two competitive methods for single-scene HSI classification, SSFTT [57] and DSNet [58], which are trained and evaluated on the same domain without explicit generalization mechanisms. All methods are evaluated using official implementations or publicly available codebases, and we carefully follow the original training protocols and hyperparameter settings to ensure fair and reliable comparisons.

For a fair comparison, all methods are trained and evaluated under exactly the same data partitioning and augmentation strategies. Specifically, considering the imbalance in sample quantities across datasets, we adopt dataset-specific data splitting: for the Pavia dataset (Pavia University as the source domain and Pavia Center as the target), 50% of the source domain data is used for training and the remaining 50% for validation; for the Houston dataset (Houston2013 as the source and Houston2018 as the target) and the HyRANK dataset (Dioni as the source and Loukia as the target), 80% of the source domain is used for training and 20% for validation. In all cases, the entire target domain is used as the test set. Furthermore, for the Houston2013 dataset, we apply data augmentation (random flip and random radiation noise) by a factor of four, which is consistently applied to all compared methods.

To account for the randomness introduced by certain modules in the compared methods, we report the classification performance using the mean ± standard deviation over multiple runs. Specifically, we fix five random seeds and compute the final results by averaging the outcomes from five independent runs using these predefined seeds. This setup ensures a more accurate and stable evaluation of all methods under consistent experimental conditions.

**Figure 10:**
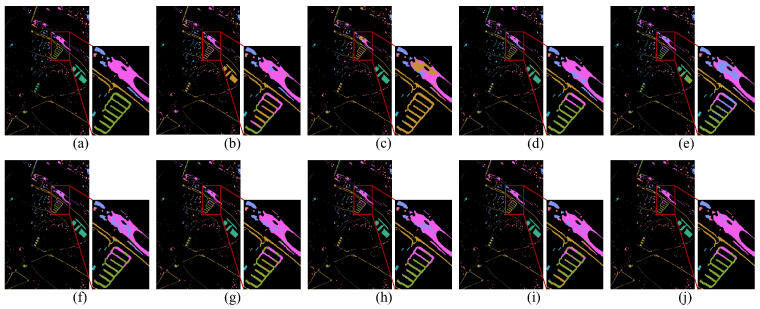
Ground truth and classification maps of different methods on the Pavia Center dataset. (**a**) Ground truth. (**b**) SSFTT. (**c**) DSNet. (**d**) DSAN. (**e**) TSTnet. (**f**) SDEnet. (**g**) FDGNet. (**h**) D3Net. (**i**) ISDGS. (**j**) Ours.

**Figure 11:**
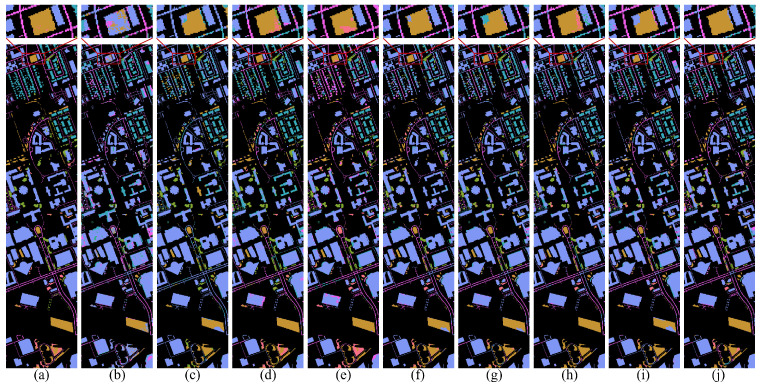
Ground truth and classification maps of different methods on the Houston2018 dataset. (**a**) Ground truth. (**b**) SSFTT. (**c**) DSNet. (**d**) DSAN. (**e**) TSTnet. (**f**) SDEnet. (**g**) FDGNet. (**h**) D3Net. (**i**) ISDGS. (**j**) Ours.

**Figure 12:**
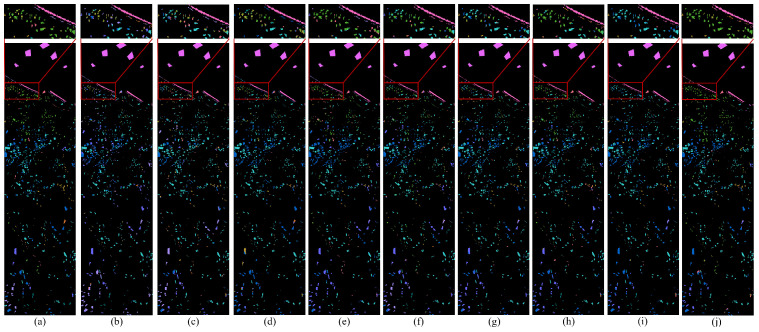
Ground truth and classification maps of different methods on the Loukia dataset. (**a**) Ground truth. (**b**) SSFTT. (**c**) DSNet. (**d**) DSAN. (**e**) TSTnet. (**f**) SDEnet. (**g**) FDGNet. (**h**) D3Net. (**i**) ISDGS. (**j**) Ours.

For DG methods, only labeled source domain data are used during training, and models are directly tested on the target domain. The same training protocol is applied to single-scene methods to assess their generalization ability under domain shift. In contrast, DA methods are trained using labeled source domain data along with an equal amount of unlabeled target domain data. Among them, DSAN requires a batch size of 32 due to its loss function design [55]. For other hyperparameters not explicitly mentioned, we follow the original settings reported in the respective papers. Table 4, Table 5 and Table 6 summarize the class-specific accuracy, OA and KC for all compared methods across the Pavia, Houston and HyRANK datasets, respectively. The visual classification results of different methods on these three datasets are illustrated in Figure 10, Figure 11 and Figure 12.

Single-scene methods (SSFT, DSNet) lack any mechanism to handle domain shift. As expected, they perform poorly when directly applied to unseen target domains. This observation further emphasizes the necessity of developing methods that explicitly address the challenges of cross-scene hyperspectral image classification.

Among DA methods, DSAN and TSTnet benefit from access to unlabeled target-domain data during training, which allows them to partially adapt to the target distribution. However, such assumptions are not applicable in the single-source domain generalization setting considered in this work.

DG methods demonstrate varying strengths across different datasets, reflecting their distinct design principles. SDEnet shows relatively stable performance across all scenes, while FDGNet performs competitively on Houston, and D3Net achieves strong results on HyRANK. These variations suggest that different design principles—such as contrastive learning or semantic alignment—impact performance under varying scene conditions. S2ECNet further explores causality-inspired design by incorporating spectral–spatial enhancement and causal contribution constraints. By introducing causal alignment through contrastive constraints on causal contribution vectors, S2ECNet exhibits strong robustness to cross-scene variations. In comparison, CauseHSI places greater emphasis on disentangling causal and non-causal features to explicitly separate invariant semantic factors from domain-specific variations. This formulation enables the model to capture stable semantic representations while flexibly accommodating domain shifts, leading to more consistent generalization across diverse scenes.

Our proposed method consistently achieves the highest OA and KC across all three datasets. Specifically, it outperforms the strongest DG baselines by clear margins on Pavia, Houston, and HyRANK, indicating superior robustness under diverse sensing conditions. The consistent improvement in Kappa further demonstrates that the performance gains are not dominated by majority classes, but reflect a more reliable agreement between predictions and ground truth.

A closer inspection of class-wise accuracies reveals that the proposed method does not uniformly improve all land-cover categories, and that performance variations across classes are clearly observable in all three datasets. In particular, several categories—such as C5 in Pavia, C1 and C4 in Houston, and C2, C6, and C10 in HyRANK—exhibit noticeable performance degradation compared with certain competing methods.

From a causal perspective, this behavior is expected rather than anomalous. The proposed framework explicitly suppresses scene-dependent and non-causal cues through causal disentanglement and counterfactual augmentation. Consequently, land-cover categories that rely heavily on background context, illumination patterns, or other scene-specific correlations—rather than intrinsic spectral–semantic properties—may experience reduced classification accuracy when such non-causal signals are attenuated.

Moreover, several degraded categories are characterized by strong spectral ambiguity or limited inter-class separability, as observed in complex datasets such as HyRANK. Under domain generalization settings, where spurious correlations cannot be exploited, learning stable causal representations for such categories remains inherently challenging for all methods. In addition, counterfactual augmentation may introduce increased variance for extremely small or noisy classes, further amplifying class-wise fluctuations.

Importantly, despite these localized degradations, the proposed method consistently achieves the highest overall accuracy and Kappa coefficient across all datasets. The improvement in Kappa indicates that the gains are not driven by a small subset of dominant classes, but instead reflect a more reliable and globally consistent alignment between predictions and ground truth under cross-scene shifts.

To further assess the reliability of the reported performance, we additionally report the 95% confidence intervals of OA and Kappa, estimated as mean $\pm t_{0.975,4}\cdot \sigma /\sqrt{5}$ using the Student’s t-distribution over five runs. As shown in Table 7, CauseHSI consistently achieves higher mean performance with relatively narrow confidence intervals across all three datasets, indicating stable and reliable performance gains.

Figure 10, Figure 11 and Figure 12 provide qualitative comparisons of classification maps. Subfigure (a) presents the ground-truth map, (b)–(i) are contrast methods, and (j) corresponds to our proposed approach. Pixels without ground-truth labels are treated as background, and all pixels are predicted for visual comparison. Notably, our method yields smoother and less noisy classification maps, as illustrated in the red-boxed regions. This visual advantage stems from the model’s focus on global semantic consistency, which reduces local misclassifications and noise.

Overall, the proposed method achieves consistent superiority in overall accuracy and domain robustness. While it may not outperform all baselines in class-level accuracy, its strong domain-invariant representation learning ensures reliable generalization across complex and diverse scenes. This further validates the effectiveness of our causal-inspired disentanglement strategy in cross-scene hyperspectral image classification.

To quantitatively assess computational efficiency, Table 8 reports training time, inference time, FLOPs, and parameter counts on three benchmark datasets. As shown in the table, CauseHSI incurs higher FLOPs than most lightweight DG baselines, with an increase of approximately 3–5× in FLOPs. However, it has the lowest number of parameters among all compared DG methods, resulting in a compact memory footprint. In terms of runtime, the training time of CauseHSI is moderately higher than that of the lightest baselines, while remaining significantly lower than heavyweight methods. Importantly, its inference time is comparable to or only marginally higher than other DG approaches across all datasets, indicating that the additional computational cost is mainly introduced during training rather than deployment. Overall, these results suggest that CauseHSI achieves a favorable balance between training-time complexity and inference-time efficiency, making it practical for real-world hyperspectral applications where robustness to domain shifts is required.

### 4.4. Ablation Study

To evaluate the effectiveness of key components in the proposed CauseHSI, we conduct ablation studies by systematically removing each component and observing performance degradation across all three datasets. The quantitative results are summarized in Table 9.

Specifically, we investigate the following six variants: (1) “no DCT”: removes the frequency-based intervention from CGM. (2) “no 2D”: removes the spectral consistency preservation branch from CGM. (3) “no Control”: disables the style-controlled discrepancy loss in CGM. (4) “no Consist&Complete”: removes both the causal consistency and completeness constraints from CDM. (5) “no Consist”: only removes the causal consistency constraint from CDM. (6) “no Complete”: only removes the causal completeness constraint from CDM.

The variants “no DCT” and “no 2D” respectively disable two complementary intervention branches in the CGM, both of which are designed to approximate counterfactual domain shifts. Removing either branch results in noticeable and consistent performance degradation across all three datasets, highlighting their synergistic roles in counterfactual generation. Specifically, the “no DCT” variant exhibits systematic drops in OA and KC, indicating that frequency-based perturbations substantially enrich the diversity of interventional samples. This empirically supports our design choice of performing counterfactual interventions in the frequency domain, as frequency components effectively capture global sensing variations such as illumination, atmospheric conditions, and sensor response, which are difficult to model explicitly through physical parameters. Similarly, the “no 2D” variant leads to clear degradation, demonstrating that preserving the central spectral structure during intervention is crucial for maintaining class-discriminative information. This confirms that counterfactual perturbations must be constrained to avoid semantic distortion, and validates the necessity of performing interventions in a controlled feature space rather than through unconstrained transformations. Moreover, the removal of the style-controlled discrepancy loss (“no Control”) causes pronounced performance drops, with HyRANK suffering the largest KC decrease (−3.30%). This observation indicates that style regulation plays a critical role in balancing semantic alignment and stylistic diversity, particularly for fine-grained land-cover categories. Without this constraint, feature-space interventions tend to over-amplify non-causal variations, reducing the utility of generated counterfactual samples. Taken together, these results provide strong empirical evidence that frequency-based intervention, spectral preservation, and style regularization play complementary roles in CGM. Frequency perturbations broaden interventional diversity, spectral preservation safeguards semantic consistency, and style control prevents over-perturbation. Their joint contribution directly supports the validity of implementing counterfactual interventions in frequency and feature spaces.

Variant “no Consist&Complete” leads to the most significant decline, as it relies solely on the causal independence constraint, failing to enforce semantic alignment or feature sufficiency. Introducing either the causal completeness (“no Consist”) or consistency (“no Complete”) constraint yields clear improvements over the basic version. Specifically, while “no Consist&Complete” shows a 3–4% drop in OA, “no Complete” reduces this drop to about 1%. These results demonstrate the importance of multi-level causal constraints in approximating true causal features.

The proposed frequency-based intervention relies on a soft frequency weighting strategy to distinguish scene-sensitive and scene-robust components in the DCT domain. Although this weighting profile is fixed across all experiments, we further investigate its sensitivity to ensure that the performance gains do not depend on a specific frequency configuration. Specifically, we vary the radial range of mid-band frequencies that receive minimal perturbation, while keeping all other components of the framework unchanged. Three configurations are considered: Narrow, Default, and Wide, corresponding to progressively smaller or larger mid-frequency preservation ranges. Notably, this variation does not introduce any explicit hard cutoff between low, mid, and high frequencies, but instead adjusts the extent of the smoothly weighted frequency regions. We evaluate these configurations on representative cross-scene classification benchmarks under the single-source domain generalization setting. As reported in Table 10, the proposed method exhibits stable performance across different frequency weighting ranges. While minor fluctuations are observed, the overall accuracy remains consistently high, indicating that the effectiveness of the proposed frequency-based intervention is not sensitive to the specific choice of frequency weighting parameters. This robustness supports our design choice of using a fixed and coarse-grained frequency weighting profile as a practical approximation of sensing-induced variations.

**Figure 13:**
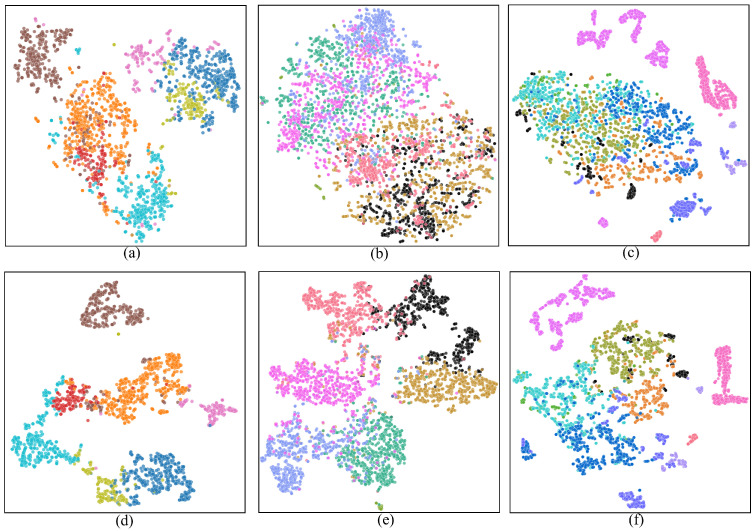
t-SNE visualization of feature distributions on three benchmark datasets. (**a**–**c**) Distributions of target-domain samples in the original feature space for Pavia, Houston, and HyRANK, respectively. (**d**–**f**) Corresponding feature distributions learned by CauseHSI on the same datasets.

To further validate the effectiveness of CauseHSI in enhancing generalization, we visualize the feature distributions on three datasets using t-SNE. Figure 13 illustrates the distributions of target-domain samples before and after applying CauseHSI. In the original feature space, samples from different classes exhibit substantial overlap, whereas CauseHSI yields clearer inter-class separation across all datasets.

### 4.5. Physical Plausibility of Generated Counterfactual Samples

Since the proposed framework relies on generated Counterfactual hyperspectral samples for DG, it is important to ensure that these samples remain physically plausible rather than arbitrary perturbations. Unlike natural images, commonly used perceptual metrics such as FID or LPIPS are not directly applicable to hyperspectral data due to the high dimensionality of spectral signals and the lack of suitable pretrained feature extractors. Therefore, we assess the physical plausibility of the generated counterfactual samples using physically grounded spectral metrics.

Specifically, we compute the spectral angle mapper (SAM) between the center-pixel spectra of the original and generated samples, where the center pixel corresponds to the semantic label in each 13×13 spatial-spectral patch. In addition, we evaluate spectral smoothness along the spectral dimension to examine whether the generated spectra preserve the inherent band-wise continuity of hyperspectral signals. As reported in Table 11, the generated counterfactual samples exhibit moderate spectral angles, typically ranging from 2.8° to 4.5°, indicating meaningful yet physically reasonable domain perturbations rather than trivial reconstructions. Moreover, the spectral smoothness of the generated samples remains close to that of real hyperspectral data across all datasets, suggesting that the proposed generation process does not introduce severe high-frequency spectral artifacts.

### 4.6. Scalability and Applicability to Large-Scale Scenes

Although the proposed framework is evaluated on commonly used benchmark datasets, it is not inherently restricted to small- or medium-sized hyperspectral scenes. The overall design of CauseHSI is patch-based and scene-agnostic, and does not rely on global scene-level modeling or full-image statistics. As a result, large-scale hyperspectral scenes can be processed in a tiled or sliding-window manner without any modification to the network architecture or training strategy.

Importantly, both the causal disentanglement module and the counterfactual generation mechanism operate locally on image patches or intermediate feature representations. Their computational and memory costs scale linearly with the number of patches, rather than with the spatial extent of the entire scene. This property ensures that the framework remains computationally feasible for large-area hyperspectral imagery.

Moreover, patch-wise training and inference are standard practice in hyperspectral image analysis, particularly for domain generalization settings where full-scene annotations are rarely available. The benchmark datasets used in this work are themselves extracted from large-scale airborne scenes, and therefore provide a representative proxy for real-world large-scene deployment. These considerations suggest that the proposed framework can be readily applied to very large-scale hyperspectral scenes in practical remote sensing applications.

## 5. Conclusions

In this work, we address the core challenge of generalizing HSI classification models to unseen domains under the single-source setting, where interventional diversity is severely limited. By adopting a causality-inspired perspective, we formulate domain shifts as interventions within a structural causal model, and propose CauseHSI, a novel framework composed of two synergistic modules. The Counterfactual Generation Module simulates diverse sensing conditions via structured frequency perturbations, enabling counterfactual sample generation that preserves semantic integrity. And the Causal Disentanglement Module disentangles causal representations from spurious domain-specific factors through a dual-branch architecture and frequency-domain reassembly. Together, these modules tackle both the lack of interventional diversity and the confounding effects of spurious correlations. We conduct extensive experiments on multiple public datasets, and the results consistently demonstrate the effectiveness of our method.

In the future, we plan to further enhance the robustness and scalability of our framework in more diverse real-world HSI scenarios. In addition, as part of future work, we plan to conduct a broader evaluation including additional DA methods to further clarify the trade-offs between generalization without target data and adaptation with target supervision. Such a systematic comparison will help delineate the complementary strengths of DG and DA approaches, thereby guiding the selection of appropriate strategies in practical applications.

## Figures and Tables

**Table 1:** Number of Source and Target Samples for the Pavia Dataset.

No.	Class	Pavia University	Pavia Center
C1	Trees	3064	7598
C2	Asphalt	6631	9248
C3	Brick	3682	2685
C4	Bitumen	1330	7287
C5	Shadow	947	2863
C6	Meadow	18,649	3090
C7	Bare soil	5029	6584
Total		39,332	39,355

**Table 2:** Number of Source and Target Samples for the Houston Dataset.

No.	Class	Houston2013	Houston2018
C1	Grass healthy	345	1353
C2	Grass stressed	365	4888
C3	Trees	365	2766
C4	Water	285	22
C5	Residential buildings	319	5347
C6	Non-residential buildings	408	32,459
C7	Road	443	6365
Total		2530	53,200

**Table 3:** Number of Source and Target Samples for the HyRANK Dataset.

No.	Class	Dioni	Loukia
C1	Dense Urban Fabric	1262	206
C2	Mineral Extraction Sites	204	54
C3	Non Irrigated Arable Land	614	426
C4	Fruit Trees	150	79
C5	Olive Groves	1768	1107
C6	Coniferous Forest	361	422
C7	Dense Sclerophyllous Vegetation	5035	2996
C8	Sparce Sclerophyllous Vegetation	6374	2361
C9	Sparsely Vegetated Areas	1754	399
C10	Rocks and Sand	492	453
C11	Water	1612	1393
C12	Coastal Water	398	421
Total		20,024	10,317

**Table 4:** Classification Results of Different Methods for the Target Scene Pavia Center Data. The 1st and 2nd best results are in bold and underlined, respectively.

No.	SSFTT	DSNet	DSAN	TSTnet	SDEnet	FDGNet	S2ECNet	D3Net	ISDGS	Ours
C1	98.76	96.59	81.24	86.36	90.16	91.79	94.18	90.77	91.87	96.33
C2	91.46	87.26	74.45	74.78	87.66	86.14	83.50	91.10	88.97	91.96
C3	20.25	57.33	79.77	61.05	72.87	75.94	70.58	75.98	70.86	75.86
C4	8.55	33.02	76.28	65.21	82.68	82.50	82.87	81.45	82.99	84.52
C5	99.78	84.21	92.73	88.85	84.18	87.91	90.85	84.06	87.49	77.56
C6	82.18	66.31	85.72	82.50	70.38	72.84	67.15	58.66	71.20	71.62
C7	88.95	75.30	77.47	66.77	82.47	83.74	85.39	85.80	86.94	84.71
OA (%)	72.12±1.25	73.11±4.32	79.18±2.03	74.60±0.63	83.73±1.89	84.54±0.91	84.13±1.12	84.27±1.39	$\underline{85.34}\;\pm \;1.48$	86.47±1.28
Kappa × 100	65.93±1.52	67.95±4.78	75.25±2.43	69.89±0.76	80.39±2.21	81.44±1.05	80.92±1.38	81.03±1.66	$\underline{82.32}\;\pm \;1.73$	83.66±1.51

**Table 5:** Classification Results of Different Methods for the Target Scene Houston2018 Data. The 1st and 2nd best results are in bold and underlined, respectively.

No.	SSFTT	DSNet	DSAN	TSTnet	SDEnet	FDGNet	S2ECNet	D3Net	ISDGS	Ours
C1	14.93	29.98	79.49	76.92	59.81	45.59	70.58	31.89	40.83	37.24
C2	28.71	42.89	70.69	74.12	76.44	76.28	74.57	80.18	75.06	82.56
C3	37.09	42.70	75.22	58.05	58.06	64.55	44.39	49.88	54.87	58.67
C4	84.54	81.82	100.00	100.00	100.00	100.00	100.00	96.36	100.00	81.82
C5	73.65	57.06	63.20	67.17	66.03	72.22	73.69	70.57	73.68	78.67
C6	78.69	91.97	77.85	83.98	88.38	89.34	87.15	89.31	88.20	91.96
C7	44.49	11.72	44.48	62.99	55.44	54.14	61.34	49.67	60.70	51.38
OA(%)	65.72±1.69	70.21±2.45	71.64±1.39	77.35±3.63	78.80±1.53	$\underline{79.81}\;\pm \;1.00$	78.92±1.41	78.33±1.23	79.31±0.95	81.78±0.36
Kappa × 100	40.60±5.67	42.68±9.32	56.36±1.56	63.93±4.22	63.90±2.44	$\underline{65.20}\;\pm \;1.55$	64.46±1.52	62.56±2.37	64.96±1.09	69.15±1.30

**Table 6:** Classification Results of Different Methods for the Target Scene Loukia Data. The 1st and 2nd best results are in bold and underlined, respectively.

No.	SSFTT	DSNet	DSAN	TSTnet	SDEnet	FDGNet	S2ECNet	D3Net	ISDGS	Ours
C1	8.42	37.09	19.25	35.34	20.51	28.74	33.50	30.39	37.38	38.93
C2	43.21	0.00	78.40	0.00	45.37	55.19	48.15	15.18	16.30	14.82
C3	36.93	74.32	35.44	37.37	31.69	60.09	43.19	41.78	38.64	65.21
C4	50.21	34.68	36.71	3.80	8.23	21.77	16.46	18.99	14.94	18.73
C5	0.00	0.40	7.71	47.51	29.02	12.36	27.37	21.82	11.62	49.99
C6	22.59	13.65	18.80	0.47	23.58	17.11	33.18	28.39	34.64	19.67
C7	75.09	83.46	74.24	72.34	76.58	79.16	81.28	78.49	75.60	64.27
C8	19.24	36.75	66.09	58.69	63.44	66.39	60.78	64.24	75.41	78.85
C9	49.96	57.34	40.69	86.82	72.87	71.78	45.86	72.78	42.10	30.13
C10	62.62	15.58	3.97	9.45	17.94	6.27	2.21	17.17	16.20	3.36
C11	98.64	100.00	100.00	100.00	100.00	100.00	100.00	100.00	100.00	100.00
C12	99.13	98.29	96.20	100.00	100.00	100.00	100.00	97.81	100.00	100.00
OA(%)	51.48±1.08	57.74±1.30	60.0±1.09	63.19±1.50	64.04±0.53	64.35±0.59	64.09±1.01	$\underline{64.61}\;\pm \;2.42$	64.34±0.68	65.46±1.12
Kappa × 100	42.45±1.78	48.50±1.57	50.39±0.80	55.25±1.89	55.95±1.09	56.00±0.93	55.76±1.22	$\underline{56.54}\;\pm \;3.10$	55.64±0.98	57.79±1.09

**Table 7:** 95% Confidence Intervals (CI) of OA and Kappa (×100) over Five Runs. For each dataset, the strongest competing method is reported for comparison.

Dataset	Method	OA (95% CI)	Kappa (95% CI)
Pavia	ISDGS	[83.50, 87.18]	[80.17, 84.47]
	Ours	[84.88, 88.06]	[81.78, 85.54]
Houston	FDGNet	[78.57, 81.05]	[63.27, 67.13]
	Ours	[81.33, 82.23]	[67.54, 70.76]
HyRANK	D3Net	[61.61, 67.61]	[52.69, 60.39]
	Ours	[64.07, 66.85]	[56.44, 59.14]

**Table 8:** Efficiency Comparison of Different Methods on Three Datasets.

Method		DSAN	TSTnet	SDEnet	FDGNet	D3Net	ISDGS	Ours
Pavia	Training time	53.84	40.31	15.75	13.85	17.40	9.21	22.96
	Testing time	13.76	6.96	6.74	7.24	8.46	6.82	8.06
	FLOPs	$1.22\times 10^{10}$	$3.53\times 10^{9}$	$2.84\times 10^{9}$	$2.43\times 10^{9}$	$4.53\times 10^{9}$	$2.07\times 10^{9}$	$1.09\times 10^{10}$
	Parameter	$2.43\times 10^{7}$	$7.70\times 10^{6}$	$4.05\times 10^{5}$	$4.90\times 10^{5}$	$5.22\times 10^{5}$	$4.65\times 10^{5}$	$3.21\times 10^{5}$
Houston	Training time	33.27	19.19	8.81	7.97	9.38	7.91	14.54
	Testing time	16.85	3.45	11.06	10.62	11.87	11.04	12.30
	FLOPs	$1.01\times 10^{10}$	$3.04\times 10^{9}$	$1.52\times 10^{9}$	$1.30\times 10^{9}$	$3.42\times 10^{9}$	$1.11\times 10^{9}$	$9.53\times 10^{9}$
	Parameter	$2.42\times 10^{7}$	$7.69\times 10^{6}$	$4.08\times 10^{5}$	$4.52\times 10^{5}$	$4.88\times 10^{5}$	$4.34\times 10^{5}$	$2.55\times 10^{5}$
HyRANK	Training time	46.64	31.37	13.13	17.49	14.76	8.07	20.92
	Testing time	5.01	3.28	2.82	3.56	3.20	3.14	3.34
	FLOPs	$1.51\times 10^{10}$	$4.19\times 10^{9}$	$4.64\times 10^{9}$	$3.98\times 10^{9}$	$6.06\times 10^{9}$	$3.40\times 10^{9}$	$1.28\times 10^{10}$
	Parameter	$2.46\times 10^{7}$	$7.73\times 10^{6}$	$4.80\times 10^{5}$	$5.36\times 10^{5}$	$5.72\times 10^{5}$	$5.01\times 10^{5}$	$3.66\times 10^{5}$

**Table 9:** Ablation Experiment of Our Method on Three Datasets.

Dataset	Metric	No DCT	No 2D	No Control	No Consist&Complete	No Consist	No Complete	Ours
Pavia	OA	85.26	84.44	84.98	82.66	84.18	85.05	86.47
	KC	82.17	81.28	81.92	79.12	81.02	81.98	83.66
Houston	OA	80.99	80.40	81.01	79.12	79.94	80.03	81.78
	KC	67.12	66.49	67.28	64.10	64.79	65.09	69.15
HyRANK	OA	64.81	64.96	63.40	61.87	62.96	63.91	65.42
	KC	56.31	57.37	54.50	52.18	53.49	55.15	57.80

**Table 10:** Sensitivity analysis of the frequency weighting strategy under different mid-band preservation ranges.

Setting	Metric	Pavia	Houston	HyRANK
Narrow	OA	85.78	80.51	65.03
	KC	82.92	68.24	58.11
Default	OA	86.47	81.78	65.42
	KC	83.66	69.15	57.80
Wide	OA	85.76	80.36	64.38
	KC	82.83	67.62	56.34

**Table 11:** Physical plausibility evaluation of generated counterfactual samples.

Dataset	Center-Pixel SAM (°)	Spectral Smoothness (Source)	Spectral Smoothness (Counterfactual)
Pavia	2.89	0.0224	0.0246
Houston	3.24	0.0327	0.0376
HyRANK	4.45	0.0544	0.0653

## Data Availability

The datasets are publicly available in Pavia at https://www.ehu.eus/ccwintco/index.php/Hyperspectral_Remote_Sensing_Scenes (accessed on 20 January 2026), Houston at https://github.com/anbydemara/CauseHSI (accessed on 20 January 2026) and HyRANK at https://huggingface.co/datasets/danaroth/hyrank (accessed on 20 January 2026).

## Abbreviations

The following abbreviations are used in this manuscript:

HSI	Hyperspectral Image
DG	Domain Generalization
CGM	Counterfactual Generation Module
CDM	Causal Disentanglement Module
CNN	Convolutional Neural Networks
SCM	Structural Causal Model
CRM	Causal Reassembly Module
DCT	Discrete Cosine Transform
SSFM	Spectral-Spatial Fusion Module
HSIC	Hilbert-Schmidt Independence Criterion
OA	Overall Accuracy
KC	Kappa Coefficient
DA	Domain Adaption
